# Surgical and Pathological Changes after Radiofrequency Ablation of Thyroid Nodules

**DOI:** 10.1155/2015/576576

**Published:** 2015-07-21

**Authors:** Chiara Dobrinja, Stella Bernardi, Bruno Fabris, Rita Eramo, Petra Makovac, Gabriele Bazzocchi, Lanfranco Piscopello, Enrica Barro, Nicolò de Manzini, Deborah Bonazza, Maurizio Pinamonti, Fabrizio Zanconati, Fulvio Stacul

**Affiliations:** ^1^UCO Chirurgia Generale, Cattinara Teaching Hospital, Strada di Fiume, 34100 Trieste, Italy; ^2^SS Endocrinologia (UCO Medicina Clinica), Cattinara Teaching Hospital, Strada di Fiume, 34100 Trieste, Italy; ^3^Department of Medical, Surgical, and Health Sciences, University of Trieste, Cattinara Teaching Hospital, Strada di Fiume, 34100 Trieste, Italy; ^4^SC Radiologia, Maggiore Hospital, Piazza dell'Ospitale, 34100 Trieste, Italy; ^5^SS Endocrinologia (III Medica), Maggiore Hospital, Piazza dell'Ospitale, 34100 Trieste, Italy; ^6^UCO Anatomia e Istologia Patologica, Cattinara Teaching Hospital, Strada di Fiume, 34100 Trieste, Italy

## Abstract

*Background*. Radiofrequency ablation (RFA) has been recently advocated as an effective technique for the treatment of symptomatic benign thyroid nodules. It is not known to what extent it may affect any subsequent thyroid surgery and/or histological diagnosis. *Materials and Methods*. RFA was performed on 64 symptomatic Thy2 nodules (benign nodules) and 6 symptomatic Thy3 nodules (follicular lesions/follicular neoplasms). Two Thy3 nodules regrew after the procedure, and these patients accepted to undergo a total thyroidectomy. Here we present how RFA has affected the operation and the final pathological features of the surgically removed nodules. *Results and Conclusions*. RFA is effective for the treatment of Thy2 nodules, but it should not be recommended as first-line therapy for the treatment of Thy3 nodules (irrespective of their mutational status), as it delays surgery in case of malignancy. Moreover, it is unknown whether RFA might promote residual tumor progression or neoplastic progression of Thy3 lesions. Nevertheless, here we show for the first time that one session of RFA does not affect subsequent thyroid surgery and/or histological diagnosis.

## 1. Introduction

Thyroid nodules are an extremely common occurrence [1]. Until recently international guidelines suggested either no treatment or surgery for patients with benign solid thyroid nodules, depending on their size, cytology, and symptoms [2]. In recent years, however, several nonsurgical minimally invasive approaches for treating thyroid nodules have been developed [3] and nowadays they represent an acceptable alternative to surgery for treating symptomatic benign thyroid nodules [3]. These techniques include percutaneous ethanol injection therapy (PEIT), laser ablation therapy (LAT), and radiofrequency ablation (RFA).

Among these techniques RFA is not only an effective [4] but also a reasonably safe [5] treatment option for recurrent cysts [6], mixed or solid benign thyroid nodules causing cosmetic concerns, local pain, or hyperthyroidism [7, 8]. Nevertheless, RFA is relatively new, so some issues regarding its outcomes still exist. For example, one of the questions that remains unanswered is what would happen if a thyroid cancer were not detected before starting RFA and was left untreated? Would RFA jeopardize a subsequent operation and/or hinder histological diagnosis?

Here we present the 2-year follow-up results of 70 patients who underwent RFA. In particular, 64 patients had Thy2 nodules (benign nodules) and 6 patients had Thy3 nodules (follicular lesions/follicular neoplasms). Because of nodule regrowth, two of these patients subsequently underwent surgery after being treated with RFA. Based on our experience the aim of this report is to describe the surgical and pathological changes after RFA of thyroid nodules and to comment on the use of RFA for Thy3 nodules.

## 2. Materials and Methods

### 2.1. Study Population

This is a retrospective study of the 2-year follow-up results of 64 patients with Thy2 thyroid nodules and 6 patients with Thy3 nodules who underwent RFA. Before the procedure, nodules were evaluated by fine needle aspiration biopsy (FNAB) twice and classified according to the British and Italian reporting systems for thyroid cytopathology [9, 10], where Thy2 corresponds to Bethesda Category II (benign nodules), and Thy3 corresponds to Bethesda Categories III and IV nodules (follicular lesions/follicular neoplasms) [11]. A single session of RFA was performed on 64 Thy2 solitary nodules, according to current recommendations [12]. A single session of RFA was also performed on 6 symptomatic Thy3 nodules in surgically high-risk individuals or patients refusing surgery. These patients did not have a family history of thyroid cancer and did not present suspicious lymph nodes. Their nodules were single, unchanged over the last three years, with no calcifications, no evidence of nuclear atypia [13, 14], and without BRAF or NRAS mutations. Patients were prepared for the procedure according to current recommendations [12] and before it they all signed an informed consent form after full explanation of the purpose and nature of all procedures used. Secondly, informed consent to use their clinical data for scientific purposes was obtained from each patient, according to the guidelines of the Azienda Ospedaliero-Universitaria di Trieste.

### 2.2. Radiofrequency Ablation Technique and Follow-Up

RFA was performed in an outpatient regimen by a well-trained radiologist experienced in US, FNAB, and RFA procedures. Before ablation, an intravenous access was obtained via an antecubital vein. Patients were placed in a supine position with their neck extended. They underwent local anesthesia at the puncture site with 2% lidocaine hydrochloride. The procedure required conscious sedation following an intravenous injection of 2-3 mg of midazolam. We used a 450 kHz radiofrequency generator (AMICA-GEN model AGN-H-1.0, HS Hospital Service SpA, Italy) delivering up to 200 W on a 50 Ω load and 18-gauge, internally cooled, monopolar electrodes (RF AMICA-PROBE model RFH18100V1, HS Hospital Service SpA, Italy) featuring a shaft length of 10 cm and an exposed tip length of either 7, 10, or 15 mm. The exposed tip length selection was based on the nodule volume: 7 mm active tips were used for the treatment of smaller nodules (<10 mL), 10 mm active tips in intermediate size nodules (10–20 mL), and 15 mm active tips in larger nodules (>20 mL).

The electrode was inserted into the thyroid nodule with a transisthmic approach under US-guidance by using a 5–18 MHz linear probe on a real-time US system (Aplio XG, Toshiba Corp. Medical Systems). To perform RFA the moving-shot technique was used [15]. The initial RF power settings ranged between 30 W and 50 W depending on the electrode exposed tip length: the shorter the active tip, the lower the power. The ablation procedure always started from the deepest areas of the nodule. If a hyperechoic zone had not formed at the electrode tip within 30 seconds, the RF generator output was progressively increased in 10 W steps, up to a maximum power ranging between 60 W and 80 W (once again depending on the active tip length). Then, once the treated zone had become hyperechoic and the electric impedance measured by the generator increased, the electrode was moved towards another area of the nodule. Ablation was terminated when transient hyperechoic zones could be identified around the entire nodule. At the end of the procedure, patients remained under observation for a couple of hours.

To evaluate RFA results, patients underwent an ultrasonography (US) and TSH measurement at baseline, 1, 3, 6, 12, and 24 months after the procedure. To simplify the calculation of the nodule volumes prior to RFA (*V*_pre_) and after RFA (*V*_post_) out of cross-sectional measurements taken from the respective US images, each nodule was approximated to an ellipsoid, both before RFA (with diameters *A*_pre_ as major axis, *B*_pre_ and *C*_pre_ as minor axis, both perpendicular to *A*_pre_) and after RFA (with diameters *A*_post_, *B*_post_, and *C*_post_, named after their pre-RFA counterparts). Therefore, the following formulas were used: *V*_pre_ = *π∗A*_pre_ *∗B*_pre_ *∗C*_pre_/6; *V*_post_ = *π∗A*_post_ *∗B*_post_ *∗C*_post_/6. The percentile volume reduction induced by the RFA treatment was calculated as Δ*V* = 100*∗*(*V*_pre_ − *V*_post_)/*V*_pre_. Technical success was deemed to be achieved when a volume reduction Δ*V* greater than 50% at 6 months from the procedure [4] was observed.

### 2.3. Surgery

Two patients were operated on after RFA. The operations were carried out by the same surgical team. The intraoperative nerve integrity monitoring (NIM) system was used in order to check the laryngeal nerve functionality. Patients, under general anaesthesia, were placed in the supine position with the neck extended. Thyroidectomy was performed by a cervicotomy of 2–5 cm in the middle area of the neck, approximately 2 cm above the sternal notch. Minimal subplatysmal flaps were created. The midline was incised and the strap muscles were retracted laterally. The vessels of the upper thyroid peduncle were selectively ligated or closed by conventional vascular clips. Special care was taken to preserve the parathyroid glands and the inferior laryngeal nerves. Once the thyroid had been removed, the area was examined for bleeding. If there was no bleeding, the incision was closed with sutures. In both cases, a surgical drain was placed to remove fluids from the area in the following days. Anaesthesia was discontinued and medication was given to wake the patient. Both patients had laryngoscopic examination for vocal cord mobility before and one month after surgery. Serum calcium levels were measured on the first and second day after surgery and weekly for the first month. Parathyroid hormone was measured 3 months after thyroid surgery.

### 2.4. Pathology

After surgery, four pathologists examined the thyroid sections stained with hematoxylin and eosin. In one case HBME-1 staining, which is considered a good marker of malignancy [16, 17], was also performed and evaluated.

Although molecular biology analyses had already been performed on the FNA of all Thy3 nodules before RFA, they were repeated on the surgically resected nodules. BRAF mutational status (including V600E/Ec, V600D, 600K, and V600R mutations) was evaluated by Therascreen BRAF RSQ PCR kit (Qiagen, UK). NRAS mutations were analysed by NRAS Pyrokit for mutations in codons 12, 13, 56, 61, 117, and 146 (Qiagen, UK). DNA was extracted from paraffin blocks and tested by using real-time polymerase chain reaction on the Rotor-Gene Q MDx 5plex HRM instrument (Qiagen, UK) and on the PyroMark Q24 System (Qiagen, UK), respectively.

### 2.5. Statistical Analysis

Continuous variables were evaluated by ANOVA. The *χ* ^2^ test was used to compare sex and nodule solidity between the two groups. The criterion for statistical significance was *p* < 0.05. All analyses were conducted with SAS (SAS Institute Inc., Cary, NC, USA).

## 3. Results

### 3.1. 2-Year Follow-Up Results after RFA

**Figure 1:**
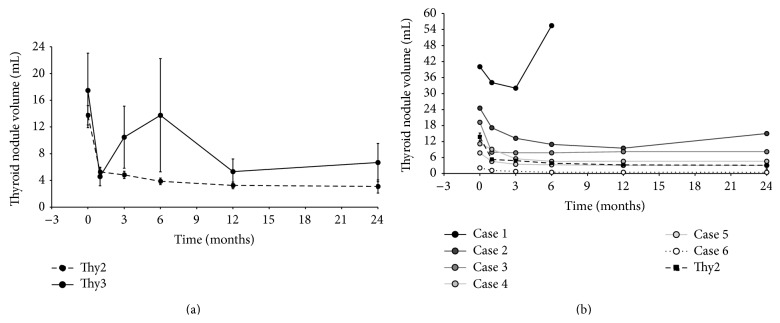
Volume (mL) reduction at 1, 3, 6, 12, and 24 months after RFA. (a) Dotted line represents Thy2 (*n* = 64) and continuous line represents Thy3 (*n* = 6) nodule volume reduction; (b) the volume reduction of each Thy3 nodule is represented together with Thy2 (*n* = 64) nodule volume reduction.

The characteristics of both groups of nodules (Thy2 and Thy3) as well as the singular characteristics of the 6 patients with Thy3 are reported in Tables 1 and 2. One session of RFA significantly reduced Thy2 nodule volume by 50%, 64%, 70%, 71%, and 67% after 1, 3, 6, 12, and 24 months from the procedure (Figure 1(a)). Technical success was obtained in all Thy2 nodules and it was maintained up to 24 months. On the other hand, even though RFA significantly reduced Thy3 nodule volume by one month (Figure 1(a)), two of these nodules regrew. In particular, after 6 months from the procedure one nodule regrew by 73% as compared to its smallest volume achieved (Figure 1(b); Case 1), while the other, after 24 months from the procedure, regrew by 57% as compared to its smallest volume achieved (Figure 1(b); Case 2). This is the reason why the curve representing Thy3 nodule volume reduction (Figure 1(a)) deviates markedly from what we observed for Thy2 nodules. Nevertheless, in the remaining four Thy3 nodules treated with RFA, the procedure significantly reduced their volume as much as what was observed in Thy2 nodules (Figure 1(b)) and this was maintained throughout all the 2-year follow-up period (Figure 1(b)). Given that nodule growth is a sign for potential malignancy [18] and that this was observed in two Thy3 nodules, where the rate of malignancy (including microcarcinomas) is estimated to be between 14 and 48% [19], the patients accepted to undergo thyroid surgery.

### 3.2. Case Presentation of Patients Who Underwent Surgery after RFA

The first patient (Case 1) is a 71-year-old man who presented with a symptomatic thyroid nodule, which was a follicular lesion (Thy3) with no evidence of nuclear atypia, no BRAF, and no NRAS mutations. Given his unwillingness to undergo surgery (in the past he had already undergone several surgical operations) and his high surgical risk (he had recently had a stroke), our multidisciplinary team [10] agreed with the patient to treat his nodule with RFA. The second patient (Case 2) is a 62-year-old woman who presented with a symptomatic thyroid nodule, which was a follicular lesion (Thy3) with no evidence of nuclear atypia, no BRAF, and no NRAS mutations. Because of her unwillingness to undergo surgery together with the stability of her nodule radiological features at the follow-up visits for more than 3 years, we agreed with the patient to treat her nodule by RFA. The characteristics of these two patients are reported in Table 2 along with the characteristics of the other Thy3 nodules treated with RFA. In the end, however, in both cases the nodules regrew, which was a sign for potential malignancy, and the patients agreed to undergo surgery.

### 3.3. Surgical Changes

In both cases, given the preoperative diagnosis of follicular lesions Thy3, BRAF, and NRAS negative, we scheduled a total thyroidectomy. Patient vocal cord mobility was normal before the procedure. The mean interval between RFA and surgery was 15 months (range 6–24). The midline incision and strap muscle retraction were feasible and in both cases the operation began from the left side, where the nodules were located, because it was the part most at risk. Remarkably, we did not find any difficulty in the ligation of the main vessels, nor in the recognition/isolation of the laryngeal nerve and the parathyroid glands, which could be always visualized. In particular, the consistency of the thyroid parenchyma was not substantially affected by RFA, and we did not see macroscopically burnt or scarred tissue. Nor did we see adhesions with the surrounding tissues. No problems were encountered in thyroid dissection. No enlarged lymph nodes were found. Total thyroidectomy was successfully completed in both cases. The mean operative time was 98.5 ± 6.5 minutes and the postoperative hospital stay was 2 days. After the operation, serum calcium levels remained within the normal ranges in both patients. No laryngeal nerve palsies or postoperative haematomas were observed.

### 3.4. Pathological Changes

**Figure 2:**
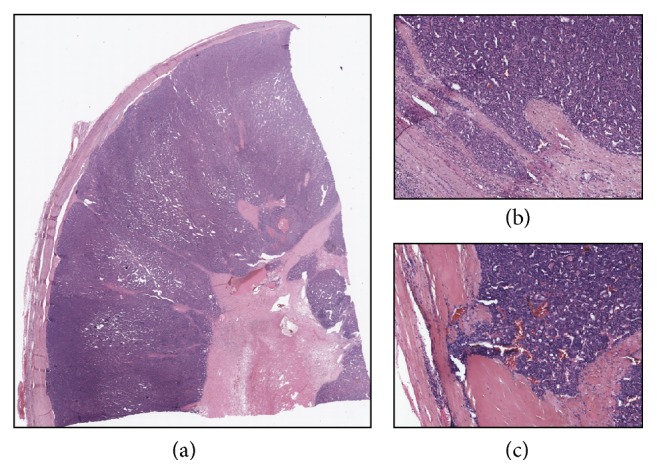
Representative images of a follicular carcinoma previously treated with RFA. (a) The lower magnification (0.8x) picture shows scattered areas of hyaline sclerosis and scarring due to RFA, which do not affect the capsule. (b-c) The higher magnification (4x and 10x, resp.) pictures show spots of capsular invasion.

**Figure 3:**
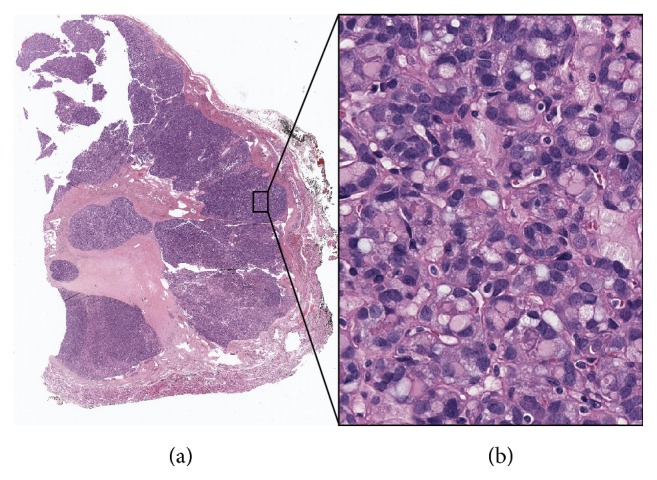
Representative images of a follicular neoplasm of uncertain malignant potential previously treated with RFA. (a) The lower magnification (0.6x) picture shows central scattered areas of hyaline sclerosis and scarring due to RFA, which do not affect the capsule. (b) The higher magnification (40x) picture shows signet ring cells, random nuclear atypia, and clearing cell changes.

The final histopathological exam showed that Case 1 was a minimally invasive follicular carcinoma (Figure 2). In particular, in the excised thyroid gland of Case 1 the left lobe was entirely occupied by a capsulated nodule measuring 6.5 × 4.5 × 3.5 cm. The cut surface showed a yellowish, heterogeneous nodule with a central area of colliquative necrosis. Microscopically the tumor was a microfollicular neoplasm with trabecular areas surrounded by a thick capsule. Central scattered areas of hyaline sclerosis and scarring, which were due to RFA, could also be seen (Figure 2). However, these areas did not alter the capsule and did not affect diagnostic assessment, since mushroom-like capsular invasion could be clearly found in a few spots (Figure 2). Therefore the final diagnosis was that of a follicular carcinoma (pT3Nx) and the patient received an ablative dose of radioiodine, with no signs of recurrence at the post thyroid ablation whole body scan. As for Case 2, this nodule turned out to be a follicular neoplasm of undeterminate malignant behaviour (Figure 3). In particular, in the excised thyroid gland of Case 2, the left lobe was almost entirely occupied by a capsulated lesion measuring 3.2 × 2.7 × 3 cm. The cut surface showed a grayish white homogeneous mass. Histopathological examination revealed that the nodule was a microfollicular neoplasm with solid and trabecular areas, surrounded by a thick capsule. Few signet ring cells, random nuclear atypia, and clearing cell changes could be identified (Figure 3). HBME-1 staining was negative. Central scattered areas of hyaline sclerosis and scarring, which were due to RFA, could also be seen (Figure 3). Again, they did not alter thyroid capsule. Since this neoplasm was well encapsulated and an exhaustive search for evidence of capsular and vascular invasion provided only suspicious or doubtful rather than definitive evidence, the final diagnosis was that of follicular neoplasm of indeterminate malignant behaviour. Of note, in both cases, BRAF and NRAS mutational status was analysed postoperatively and, consistent with our previous results, we did not find any mutations.

## 4. Discussion

Our study confirms the efficacy and applicability of current guidelines, indicating that RFA can be used effectively to treat benign thyroid nodules, cytologically defined as Thy2 nodules. In fact, RFA significantly reduced the volume of all Thy2 nodules and this was maintained throughout the study period [8]. This is consistent with previous studies demonstrating that RFA can reduce nodule volume by 33–58% after one month, by 51–85% after six months, and by 93% after four years from the procedure [3].

On the other hand, our data demonstrates that RFA should not be recommended as first-line therapy in Thy3 nodules, whose malignancy rate is between 14 and 48% [19]. This report shows that in 2 patients out of 6 (33%) with Thy3 nodules, which turned out to be cancers, RFA not only failed to resolve the patients' problem, but it also delayed surgery, which is the recommended curative approach for thyroid cancer [10]. While RFA is an effective and safe alternative to repeat surgery in patients with locally recurrent small thyroid cancers [20, 21], it is not yet recommended in patients with thyroid cancer who have not had previous total thyroidectomy and/or neck dissection [22]. Larger randomized studies might demonstrate the long-term efficacy of RFA compared to surgery for the first-line treatment of thyroid cancer.

Taking into account the rapid regrowth of the nodule where we found a follicular carcinoma (Figure 1(b)), it should be evaluated whether RFA might promote neoplastic cell transformation or the progression of undetected thyroid cancers. This is an important issue to address before recommending RFA for Thy3 nodules, since these nodules are considered premalignant precursors of follicular carcinoma [23], if they are not already a cytologically undetected thyroid cancer [19]. A few reports have already raised the issue of tumor regrowth in thyroid [24] as well as nonthyroid cancers [25, 26]. As for thyroid cancer regrowth, Park and colleagues found that 2 of the 9 recurrent thyroid cancers treated with RFA regrew after a follow-up of at least 6 months from the treatment [24]. Notably, in our cohort (as well as in the cohort of Park) the nodules with a volume greater than 20 mL were those that regrew. So although the factors that might have caused thyroid tumor regrowth remain unclear, it is possible that heat trauma as well as tumor size and behaviour might have influenced nodule regrowth.

In hepatocellular carcinomas (HCC), for example, rapid tumor progression and sarcomatous changes have been reported after RFA [25, 26]. These events are believed to be due to the progression of residual HCC, as it has been demonstrated that insufficient RFA facilitates rapid progression of residual HCC [27], where suboptimal heat promotes angiogenesis via hypoxia-inducible factor-1*α* and vascular endothelial growth factor-A [28]. Moreover, it has also been shown that even nontumor cells, such as tumor-associated endothelial cells, which are exposed to RFA, change their behaviour once exposed to insufficient RFA. In particular, if these cells are not completely coagulated when exposed to suboptimal (lower) temperatures, ranging from 42°C to 47°C, they exhibit enhanced angiogenesis and promote the invasiveness of residual hepatocellular carcinomas [29]. In addition to that, Breitenbuch et al. showed that RFA enhanced the proliferation of distant, preexisting, undetected, residual single neoplastic cells, while destroying completely the primary liver neoplasm [30]. This effect was attributed to immunological and biological consequences of heat trauma and was consistent with the report by Solbiati et al. that 57% of patients treated with RFA developed new distant metastases afterwards [31].

Nevertheless, our study also shows that in the remaining 4 patients (67%) with Thy3 nodules, RFA significantly reduced thyroid nodule volume and this was maintained up to 2 years, as in Thy2 nodules. Unfortunately, it is not possible to determine if the Thy3 nodules that responded did not regrow because they were benign or because they harboured small neoplastic foci that have been entirely destroyed by RFA, as happened in a group of microcarcinomas treated by PLA [32]. It could be speculated that since the nodules that responded to RFA were small lesions, we did not see any regrowth there because they were entirely destroyed by the procedure and no residual tissue was left. Therefore, before recommending RFA for the treatment of Thy3 nodules, we believe that further studies are needed: to exclude the risk that this technique might significantly facilitate transformation of follicular lesions into follicular carcinomas or promote the progression of residual thyroid cancers, as well as to clarify whether, on the other hand, it can be safely applied to Thy3 nodules regardless of/or depending on their size.

Having said that, we believe this study clearly demonstrates that RFA does not affect subsequent thyroid surgery and/or histological diagnosis. Based on our experience, the surgical removal of thyroid nodules is not impacted by one session of RFA, as the patients who had undergone this procedure could be successfully operated on with no complications. In the thyroid, few data [33] exist on the operative and pathological findings in patients having undergone minimally invasive nonsurgical techniques. In the liver, on the contrary, we know that RFA leads to the formation of a fibrous capsule around an area of coagulative necrosis [34, 35]. For this reason we were concerned that RFA could have induced fibrosis of the structures surrounding the nodule and the thyroid, which actually did not happen. This could be ascribed to our technique, since we are used to keeping the active tip of the needle within the nodule all through the procedure, which allows us to undertreat the areas adjacent to the capsule and to the laryngeal nerve in order to prevent possible complications, such as nerve injury. For the same reason we believe the procedure did not affect the histopathological examination of the thyroid. In the excised thyroid glands, central and rather scattered areas of hyaline sclerosis and scarring could be noted, which did not alter the capsule and the tissue sufficiently to alter the diagnostic assessment. In both cases the features of microfollicular neoplasms could be identified and in one case we found some spots of capsular invasion, leading us to the identification of a follicular carcinoma.

In conclusion, RFA is effective for the treatment of Thy2 nodules, but in our experience it should not be recommended for the treatment of Thy3 nodules (irrespective of their mutational status), as it delays surgery in case of malignancy. Moreover, further studies are needed to clarify whether RFA might facilitate the transformation of follicular lesions to follicular carcinomas and/or residual thyroid tumor progression and therefore whether it can be safely applied to the treatment of Thy3 nodules. Nevertheless, here we show for the first time that one session of RFA does not affect subsequent thyroid surgery and histological diagnosis.

## Figures and Tables

**Table 1:** 

Characteristics	Thy2 (*n* = 64)	Thy3 (*n* = 6)	*p* value
Age (years)	60.47 ± 1.89	61.16 ± 5.62	n.s.
Sex (M)	17	3	n.s.
Nodule max diameter (mm)	36.04 ± 2.40	38.17 ± 4.12	n.s.
Nodule volume (mL)	13.81 ± 1.86	17.46 ± 5.57	n.s
Solidity > 50%	55	6	n.s
Solidity < 50%	9	0	n.s.
BRAF mutations	Not tested	Absent	—
NRAS mutations	Not tested	Absent	—
TSH (*μ*U/mL)	1.35 ± 0.23	1.41 ± 0.47	n.s.
Calcitonin (pg/mL)	1.62 ± 0.14	1.42 ± 0.36	n.s.

**Table 2:** 

Characteristics	Case 1	Case 2	Case 3	Case 4	Case 5	Case 6
Age (years)	71	60	50	53	79	42
Sex	M	F	F	F	M	M
Nodule max diameter (mm)	53	42	41	38	32	23
Nodule volume (mL)	40	24	19	11	7	2
Type of nodule	Solid nodule	Solid nodule	Solid nodule	Solid nodule	Solid nodule	Solid nodule
FNAB	Thy3	Thy3	Thy3	Thy3	Thy3	Thy3
BRAF mutations	Absent	Absent	Absent	Absent	Absent	Absent
NRAS mutations	Absent	Absent	Absent	Absent	Absent	Absent
TSH (*μ*U/mL)	1.38	1.94	1.22	0.65	0.02	3.35
Calcitonin (pg/mL)	1.3	1	1	1	1	3.2
Anti-TPO and/or Anti-TG Abs	Absent	Absent	Absent	Absent	Absent	Absent
